# The practice of advance care planning among ambulatory cancer patients at a tertiary hospital in Kenya: descriptive cross-sectional survey

**DOI:** 10.3332/ecancer.2025.1926

**Published:** 2025-06-12

**Authors:** Lavender Otom, Peter Oyiro, Elijah Ogola

**Affiliations:** 1 Jaramogi Oginga Odinga Teaching and Referral Hospital, PO Box 849-40100, Kisumu, Kenya; 2 Department of Clinical Medicine and Therapeutics, University of Nairobi, PO Box 30197-00100, Nairobi, Kenya; a https://orcid.org/0009-0003-4105-906

**Keywords:** advance care planning, advanced cancer, advanced care directives, surrogate decision maker, living will, palliative care

## Abstract

**Background:**

Advance care planning (ACP) is relevant in the care of cancer patients. Insight into the current practice of ACP can identify high-priority areas to direct interventions aimed at improving the process.

**Objective:**

To assess the practice of ACP among ambulatory cancer patients at a Kenyan hospital.

**Methodology:**

A descriptive cross-sectional survey was conducted at the ambulatory oncology clinic at a tertiary referral hospital. We recruited 387 study participants through consecutive sampling among heterogenous cancer patients. An interviewer-administered questionnaire was used to collect data, which was analysed by SPSS version 25 and through multivariable logistical regression.

**Results:**

Among 387 participants, 78.55% were females. The uptake of advance directives was low; only 27.13% of participants had appointed surrogate decision makers, while 1.5% had living wills. Few had discussed end-of-life wishes with family (28.68%) and doctors (19.63%). Only 27.39% had discussed life expectancy with a doctor. Among those who had not participated in ACP, most were willing to discuss life expectancy (71.9%); discuss end-of-life wishes with family (81.2%) and doctors (85.1%); complete advance directives (68%) and appoint surrogate decision makers (75.9%) in the next 1 month. Doctors were most preferred to initiate ACP discussions. Factors that positively correlated with uptake of advanced directives (ADs) included – ECOG status, discussion with family and with doctors.

**Conclusion:**

The uptake of ADs among ambulatory cancer patients was low; additionally, self-reported participation in ACP was low. Our study highlights the need for widespread education initiatives and standardisation of the ACP process.

**Recommendations:**

There is a need for further studies and strategies to improve the participation in ACP and hence the quality of life among patients with malignancies in Kenya.

## Introduction

Advance care planning (ACP) is an iterative process through which adults at any stage of health have conversations with healthcare workers that aid in understanding their values, preferences and goals regarding future medical care and thus align healthcare provision with these goals and preferences [1]. The processes of ACP may lead to documentation of these preferences and instructions in legal documents known as advance directives, with the healthcare proxy designation/surrogate decision maker and the living will serving as the primary instruments for documentation of advance directives. Advance directives are affected only when a person becomes incapacitated and thus unable to make decisions on their own [1].

Undertaking ACPs confers benefits to the individual, the family and the health care system. In the case of the individual, these include increasing a person’s autonomy, receiving treatment based on the person’s preferences, improved quality of life and higher satisfaction with the level of care at the end of life as well as reducing the healthcare costs at the end of life. For family members, it decreases their decisional burden, and eases stress, anxiety and depression experienced after death among other outcomes. It also decreases healthcare workers’ moral distress and ethical dilemmas encountered in end-of-life care [2].

From population studies, the prevalence rates of ACP and advance directives have varied from 36.7% in the United States [3], 29.8% in Australia [4], 10% in Germany and 2% in Spain [5]. The prevalence rates of ACP among African Americans have been lower than those reported among white Americans (17%–24% versus 30%–45%) [6]. No data on the uptake of ACP in Africa is currently available. Several studies have looked at the prevalence of ACP and advanced directives (ADs) in the context of patients with a diagnosis of cancer. Bar-Sela *et al* [7] noted that 45% of the study population in an outpatient cancer unit in Israel had completed ACP documents in a cross-sectional survey. Waller *et al* [8] in a cross-sectional survey reported that only 11% of oncology outpatients in an Australian setting had discussed their wishes with a doctor, despite 66% of those who had not engaged in ACP discussions reporting that they were willing to do so. To date, there has been only one study on the utilisation of advance directives among terminally ill patients at a tertiary facility in Kenya. The findings revealed an uptake of advance directives in this context, with a rate of 41.2% [9].

The relevance of ACP in the African context was highlighted by the findings of a qualitative study conducted in South Africa: ‘A vast majority of the study participants agreed that ACP was relevant in their local context including formalising conversations and preferences through living wills and appointing proxy decision makers’ [10].

Cancer ranks as the third leading cause of death in Kenya, with about 42,116 new cases and 27,092 cancer deaths recorded in 2020 [11]. Approximately 68% of cancer cases in our setup are diagnosed late because of ignorance, shortage of adequate facilities to aid in the diagnosis, the high cost of medical services, including diagnostic as well as treatment facilities and high poverty levels [12]. Cases of cancer are expected to rise by more than 120% in the next 20 years [11]. Despite this, there is a paucity of data on the practice of ACP and the uptake of advance directives among cancer patients. The unmet need for palliative care remains huge, with only 14,552 Kenyans accessing these services of the 800,000 in need of it [13]. These aspects indicate a need to understand the practice of ACP among cancer patients in a bid to develop culturally sensitive initiatives that could improve uptake and aid in realising the benefits associated with ACP processes in the context of a low-resource set-up.

This study assessed the practice of ACP and determined the uptake of advance directives among ambulatory cancer patients at a Kenyan hospital.

### Objectives

The study was carried out to assess the practise of ACP and uptake of advance directives among ambulatory cancer patients at a tertiary teaching and referral hospital.

Specific objectives were (1) to determine the proportion of patients who had adopted advance directives among ambulatory cancer patients, and (2) to evaluate the proportion of ambulatory cancer patients participating in ACP discussions.

## Methods

### Study design and setting

A cross-sectional descriptive survey was utilised. The study was conducted in a tertiary teaching and referral hospital in Kenya. The hospital is the largest public regional referral hospital that offers comprehensive cancer care services at subsidised rates. It has an inpatient bed capacity of over 1,800 and provides outpatient services to approximately 34,000 cancer patients annually. Data were collected from the period of September to November 2022.

### Sample

The sample size was determined using Cochran’s formula for descriptive studies [14]: *n* = (Z2 × P × (1 – P))/e2. Based on the findings of a systematic review, an estimated proportion of patients with advance directives of 36.7% was used [3]. The final sample was estimated at 328/330 participants. After further adjustment of 10% to account for ineligible participants, the final sample was 363 participants. Ultimately, we recruited 387 participants for this study.

### Sampling technique

Consecutive sampling was applied to recruit the study participants. Eligible participants were identified using clinic lists. Participants were recruited by the research team while waiting in line. The aim of the study and sampling process was adequately communicated during the sensitisation health talks at the various clinics. This also helped avoid double recruitment. Once the participant was selected, their file was marked with an orange sticker and for those who had participated or were not selected, their files were marked with a green sticker. Consecutive sampling was repeated daily from Monday to Friday for 3 months. On average, 4 participants were enrolled per day until the targeted sample size of 387 was reached.

### Inclusion criteria

The patients were recruited to participate in the study if they had a confirmed diagnosis of cancer. They were either on cancer treatment or on follow up after completion of treatment. All were adults aged 18 years and above and had given informed written consent and were able to read, write and speak English or Kiswahili. Patients with dependent functional status or those with psychiatric disorders were excluded from the study.

### Recruitment

Participants who met the inclusion criteria were approached by the first author at the cancer treatment centre. The purpose and procedures of the study were explained. Written informed consent was obtained from the patients.

### Data collection

An interviewer administered a questionnaire, which was the main study tool for this survey. It was used to obtain data on the perceived importance of participating in ACP activities, willingness to participate in ACP activities in the next 1-month, self-reported participation in ACP activities and on who should initiate ACP discussions.

Demographic data, such as age, marital status, level of education and employment status, were collected with a survey questionnaire. Clinical information, such as cancer staging and type of cancer, treatment information, were extracted from medical records.

The data collection tool was adopted with permission from a study by Waller *et al* [8] based in a medical oncology outpatient setting in Australia and was available in English and Kiswahili. It contained five sections: section A which had one statement on self-reported health status in the past week, section B with four statements on the perceived importance of participating in ACP activities, section C with five statements on willingness to participate in ACP in the next month, section D with five statements on self-reported participation in ACP and section E with four statements on who should initiate ACP discussions. The tool was validated for use in our setup with a Cronbach’s reliability coefficient of 0.85.

### Data analysis

Data analysis was conducted using SPSS version 25. Descriptive statistics, such as mean with standard variation, frequencies and percentages, were used to summarise the respondents’ baseline characteristics. Frequencies and percentages were used to assess the proportion of patients who had adopted advance directives as well as the proportion of ambulatory cancer patients participating in ACP discussions. Pearson chi-square test of association and multivariable logistic regression were used to assess the association between respondents’ characteristics and uptake of advance directives. Statistical significance was considered at a *p* < 0.05.

### Ethical considerations

Ethical approval for this study was granted by the University of Nairobi Department of Clinical Medicine and Therapeutics and the KNH-UoN Ethics Review Committee (Reference number P435/05/2022). The informed consent form was signed before data collection. Confidentiality and privacy were assured throughout the study by maintaining the anonymity of the participants and storing the data in password-protected files.

## Results

### Sociodemographic and clinical characteristics

The average age of the study participants was 51.6 (SD ± 13.7) years. The majority were females, 78.5% (304/387); more than half (58.7%) resided in rural areas. Only 61(16.5%) had a tertiary level of education and 381 (98%) were Christians; 249 (64.3%) were unemployed Table 1.

The most common cancer type was breast cancer (31.0%), followed by cervical cancer (22.5%). Early-stage cancer (stages 0, 1 and 2) constituted 47.8% at diagnosis, while 22.7% had locally advanced disease at diagnosis, with 22.0% having had metastatic disease at diagnosis. Overall, 28.4% of participants had been diagnosed with cancer less than 6 months at the time we conducted the interviews, while 24.8% of participants had been diagnosed more than 2 years prior to the time we interviewed them. The majority (60.0%) were ECOG functional status 1, while 21.2% of the participants were at ECOG 0. Table 2 summarises the participants’ clinical characteristics.

### Uptake of advance directives

Overall, 105 (27.1%) of the participants had appointed a surrogate decision maker, while only 6 (1.5%) had documented their end-of-life wishes in a written document (an advance directive) (Table 3). Table 4 depicts Self-reported participation in ACP activities. Altogether, 111 participants (28.7%) had already discussed the type of end-of-life care they would want to receive with their family; however, only 19.6% had discussed this with their doctor. In total, 200 participants (51.7%) had not participated in any ACP activity.

Table 5 summarises participants’ reported views on the importance of participating in ACP activities. Most of the participants strongly agreed that it was important to discuss end-of-life wishes with family (89.4%) and with a doctor (91.0%). Fewer participants strongly agreed that it was important to record end-of-life wishes in a written document, i.e., an advance directive (67.4%).

Table 6 depicts participants’ willingness to participate in ACP activities. Among participants who had not participated in each ACP activity, 81.2% wanted to talk to their family about the type of end-of-life care they wanted to receive. Fewer participants (68%) wanted to record the type of end-of-life care in an advance directive. On whom was most suited to initiate ACP discussions, 70.9% of study participants who responded strongly agreed that the doctor should initiate ACP discussions, while 57.4% of respondents strongly agreed that the patient was best suited to initiate ACP discussions (Table 7).

The following factors were noted to positively correlate with the uptake of advance directives (*p* < 0.05) on multivariable analysis: ECOG status 1-2, prior discussions with family and doctors.

### ACP documentation in participants medical records

**Figure 1.**
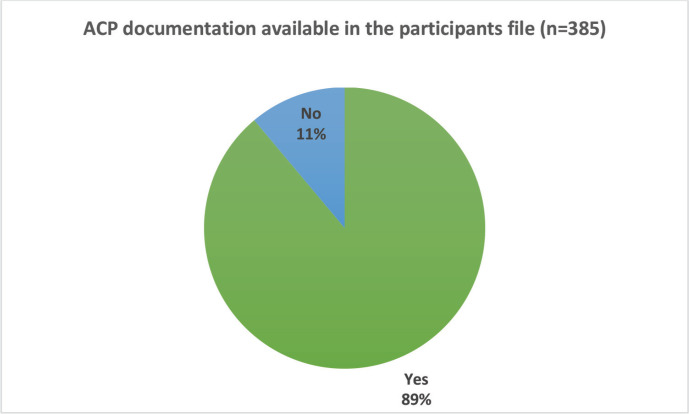
Proportion of patients with advanced care plan documented in the file.

Of the 387 participants, we found that 88.9% had evidence of documentation of ACP discussions in their files (Figure 1); these were in the form of the Clinical Navigation tools and doctors’ documentation of family conference discussions.

We did not find any completed official hospital family conference tool, nor surrogate decision maker appointment orders or a living will in any of the files that we audited.

## Discussion

ACP is increasingly recognised as standard care in the spectrum of cancer management and has been incorporated in practice guidelines and policy documents [13, 15, 16]. This study was a descriptive cross-sectional survey to determine the practice of ACP among a heterogenous population of ambulatory cancer patients in a low-resource setup.

We found that a very small proportion of study participants had adopted advance directives, with less than one third (27.1%) having appointed surrogate decision makers and only 1.5% completing living wills. Waller *et al* [8] in a heterogenous population of cancer outpatients found that 28.1% had formally chosen surrogate decision makers, a proportion that was almost similar to our study population. They also noted that 15.1% of their study population had recorded an advance directive [8]. Our findings may be in part due to the shift in focus in the field of ACP from the completion of ADs to a greater focus on having patients hold more comprehensive ACP discussions with their health care workers and aid them in understanding their values, preferences and goals of care [1, 8]. The reliance of our study on recall may have also negatively affected our findings of the uptake of advance directives.

The low uptake of living wills may be related to a lack of national laws governing advance directives as well as the lack of an institutional policy that would lay a framework for healthcare workers to guide patients in the completion of these documents [9, 17]. In comparison to the other domains of ACP, of those participants who had not participated in ACP activities, fewer participants (68%) were willing to document their end-of-life wishes in an advance directive. It is possible that cultural factors played a role in this finding. Cultural influence contributing to low uptake of living wills was seen in a qualitative study by Collins *et al* [18] among African Americans where faith in God and belief in life after death as well as the fear of talking about death emerged as the main themes among their informants and diminished the importance of written documents.

Across all the domains that we interrogated, there was a preference to have family involvement in ACP. We noted that 28.7% of study participants had talked to family about the type of end of life wishes majority of whom had appointed surrogate decision makers, and it is thus possible that their surrogate decision makers were family members although our study did not specifically review the relationship of the surrogate decision maker to the study participants. Bar-Sela *et al* [7] noted that not having a close enough relative who would make decisions on a patient’s behalf negatively impacted the completion of ACP documents in an Israeli outpatient cancer centre [7]. Collins *et al* [6] in an integrative review of literature to determine cultural aspects of end-of-life care planning among African Americans noted that African Americans were more likely to depend on family members and trusted clergy to communicate their preferences for end-of-life and our findings seem to be in accord with this.

More participants who had not engaged in ACP were willing to participate in ACP discussions in the next 1 month compared to what Waller *et al* [8] found (85.1% were willing to discuss with a doctor versus 57%, 68% were willing to document an advance directive versus 56% and 75.9% were willing to appoint surrogate decision makers versus 40% in the study by Waller *et al* [8]). There was a lower preference for living wills in our setup relative to what Waller *et al* [8] found in the Australian population. This finding may have an impact on the allocation of resources in the implementation of ACP in our setup, as more resources may be directed towards advocating for doctor-led ACP discussions and surrogate decision maker appointments while seeking ways to improve documentation of advance directives.

Factors that positively correlated with the uptake of advance directives included: the ECOG functional status, having a discussion with family and having a discussion with a doctor. Similar to what Omondi *et al* [9] found in a population of terminally ill patients, participants who had discussed preferences of end-of-life care with their family were more likely to have completed an Ads. A discussion with the doctor on participants’ end-of-life wishes may have meant that they got an opportunity to acquire knowledge on the ACP process, which may have culminated in the completion of advance directives. Participants who were more stable at ECOG 1-2 were more likely to have AD and this raises the question whether clinicians deferred ACP discussions with sicker patients with the notion that these discussions may negatively impact their outcomes. It has been previously seen that clinicians tend to withhold ACP discussion for sicker patients and thus may serve as a limitation for the uptake of ACP in such patients who may benefit most from them [19].

Our findings were not in accord with those of Omondi *et al* [9] who noted that greater functional impairment was associated with a higher uptake of ACP, although the differences in our study populations may explain the differing findings. Omondi *et al* [9] study was among terminally ill patients, and thus clinical teams held ACP discussions with the families in most cases, as opposed to this ambulatory population.

There is a need to conduct further assessment and institute measures like education of clinicians to facilitate ACP discussions and introduction of advance directives across all patient groups regardless of health status. We did not, however, find any association between uptake of AD and age, level of education and religion; factors which have been seen to be associated with the uptake of AD in other studies [20].

## Conclusion

The uptake of advance directives among ambulatory cancer patients is low despite recognition of its benefits in end-of-life care. There was a preference for the appointment of surrogate decision makers over the completion of living wills. Enhanced participation of doctors and family in ACP may improve the uptake. Cultural influences may have played a role in the preferences for more family-centred ACP practices and should be explored further.

Factors that were associated with uptake of AD mirrored those from past studies, although age, level of education and religion were not associated with uptake of AD as seen in Western studies.

Early initiation of ACP and clear communication of its implications in the spectrum of cancer is a high-priority area in our setup and may help mitigate the crisis situations encountered in end-of-life care.

## Limitations

This research study was quantitative by design; mixed-method designs with a larger sample size are recommended in future studies. In addition, the cross-sectional design of this study may not allow us to infer a causal relationship between sociodemographic characteristics and types of malignancies and uptake of advance directives. The reliance on recall for the data on the domains of ACP may have impacted negatively on the results we obtained. Data collected on the uptake of AD was also not verifiable. Our sample constituted a heterogenous population of cancer patients at different disease stages and on different treatment strategies and may not be generalisable to a specific demographic of cancer patients, e.g., patients with advanced cancer.

## Conflicts of interest

The authors declare that there are no conflicts of interest.

## Funding

This study was self-funded. There was no external funding.

## Author contributions

Conceptualisation: LO, with PO and EO’s supervision; Literature search: LO, with PO’s supervision; Study design: LO, with PO and EO’s supervision; Data collection: LO, with PO and EO’s supervision; Data analysis: LO, with PO and EO’s supervision; Manuscript preparation: LO, with PO’s supervision; Writing and editing of manuscript: LO, with PO and EO’s supervision. All authors read and approved the final version of the manuscript.

## Figures and Tables

**Table 1.** Sociodemographic characteristics.

Variable	Category	Number (*n* = 387)	Percent (%)
Age (in years)	Mean (SD)	51.6 (13.7)	
	Median (IQR)	50 (43–62)	
	Range	18–92	
Gender	Male	83	21.5
	Female	304	78.5
Marital status	Single	54	13.95
	Widowed	47	12.14
	Divorced	28	7.24
	Married	258	66.7
Highest level of education	No formal education	12	3.1
	Primary	164	42.4
	Secondary	150	38.8
	Tertiary	61	15.8
Religion	Christian	381	98.4
	Muslim	5	1.3
	Traditional	1	0.3
Employment status	Employed	38	9.8
	Self employed	86	22.2
	Unemployed	249	64.3
	Other	14	3.6
Residence	Urban	160	41.3
	Rural	227	58.7
Estimated monthly income (KES)	<5,000	219	56.6
	5,000–10,000	73	18.9
	10,001–25,000	58	15.0
	25,001–50,000	32	8.3
	>50,000	5	1.3

**Table 2.** Clinical characteristics of study participants.

Variable	Category	Number	Percentage (%)
Diagnosis (*n* = 387)	Breast cancer	120	31.0
	Cervical cancer	87	22.5
	Oesophageal cancer	22	5.7
	Colorectal cancer	20	5.2
	Lymphoma	16	4.1
	Prostate cancer	15	3.9
	Nasopharyngeal carcinoma	10	2.6
	Gastric cancer	10	2.6
	Others	86	22.2
	I don’t know	1	0.003
Months since diagnosis	<6 months	110	28.4
	≥6–12 months	111	28.7
	≥12–24 months	70	18.1
	≥24 months	96	24.8
Stage at diagnosis	Early	185	47.8
	Locally advanced	88	22.7
	Metastatic disease	85	22
	Not documented	29	7.5
Performance status (ECOG)	0	82	21.2
	1	232	60.0
	2	60	15.5
	3	11	2.8
	4	2	0.5
How well do you understand your cancer	Not at all	38	9.8
	A little	87	22.5
	A little more	79	20.4
	Averagely	110	28.4
	Well	54	14.0
	Very well	19	4.9

**Table 3.** Uptake of advance directives.

ACP activities: have already	Yes	No	Unsure
	***N* (%)**	***N* (%)**	***N* (%)**
Recorded an advance directive	6 (1.5)	380 (98.2)	1 (0.3)
Formally chosen someone to make decisions about your care on your behalf (i.e., a surrogate decision maker)	105 (27.1)	280 (72.4)	2 (0.5)

**Table 4.** Self-reported participation in ACP activities.

ACP activities: have already	Yes	No	Unsure
	***N* (%)**	***N* (%)**	***N* (%)**
Talked with your family about the type of end-of-life care you would want to receive	111 (28.7)	274 (70.8)	2 (0.5)
Talked with your doctor about the type of end-of-life care you would want to receive	76 (19.6)	309 (79.8)	2 (0.5)
Discussed how cancer may affect the length of your life (your life expectancy) with your doctor	106 (27.4)	267 (69.0)	14 (3.6)

**Table 5.** Patients views about the importance of participating in each of the ACP activities.

ACP activities	Strongly agree	Somewhat agree	Somewhat disagree	Strongly disagree
	***N* (%)**	***N* (%)**	***N* (%)**	***N* (%)**
Talk to your family about the type of end of life care you would want to receive	346 (89.4)	10 (2.6)	17 (4.4)	14 (3.6)
Talk to your doctor about the type of end of life care you would want to receive	352 (91.0)	11 (2.8)	12 (3.1)	12 (3.1)
Record the type of care you would want to receive in a document, i.e., an advance directive)	261 (67.4)	25 (6.5)	70 (18.1)	31 (8.0)
Formally choose someone to make decisions about your care on your behalf (i.e., a surrogate decision maker)	331 (85.5)	13 (3.4)	26 (6.7)	17 (4.4)

**Table 6.** Willingness to participate in ACP activities.

ACP activities	Yes	No	Unsure	Total
	*n* (%)	*n* (%)	*n* (%)	*N*
Talk to family about the type of end-of-life care s/he would want to receive	224 (81.2)	38 (13.8)	14 (5.1)	276
Talk to doctor about the type of end-of-life care s/he would want to receive	265 (85.1)	33 (10.7)	13 (4.2)	311
Record the type of care s/he would want to receive in a written document	259 (68.0)	95 (24.9)	27 (7.1)	381
Formally choose someone to make decisions about care on his/her behalf	214 (75.9)	46 (16.3)	22 (7.8)	282
Discuss life expectancy with his or her doctor	202 (71.9)	57 (20.3)	22 (7.8)	281

**Table 7.** Participants views on who should initiate ACP discussions.

	Strongly agree	Somewhat agree	Somewhat disagree	Strongly disagree	Did not respond	Total
	*n* (%)	*n* (%)	*n* (%)	*n* (%)	*n* (%)	*N* (%)
Patient	210 (54.3)	76 (19.6)	13 (3.4)	67 (17.3)	21(5.4)	387(100)
Family	81 (20.9)	118 (30.5)	21 (5.4)	134 (34.6)	33(8.5)	387(100)
Doctor	266 (68.7)	43 (11.1)	10 (2.6)	56 (14.5)	12(3.1)	387(100)
Other	12 (3.1)	2 (0.5)	0(0)	201 (51.9)	172(44.4)	387 (100)
